# History-Dependent Excitability as a Single-Cell Substrate of Transient Memory for Information Discrimination

**DOI:** 10.1371/journal.pone.0015023

**Published:** 2010-12-28

**Authors:** Fabiano Baroni, Joaquín J. Torres, Pablo Varona

**Affiliations:** 1 Grupo de Neurocomputacion Biologica, Departamento de Ingeniería Informatica, Escuela Politecnica Superior, Universidad Autonoma de Madrid, Madrid, Spain; 2 Departamento de Electromagnetismo y Fisica de la Materia and Institute Carlos I for Theoretical and Computational Physics, Universidad de Granada, Granada, Spain; Mount Sinai School of Medicine, United States of America

## Abstract

Neurons react differently to incoming stimuli depending upon their previous history of stimulation. This property can be considered as a single-cell substrate for transient memory, or context-dependent information processing: depending upon the current context that the neuron “sees” through the subset of the network impinging on it in the immediate past, the same synaptic event can evoke a postsynaptic spike or just a subthreshold depolarization. We propose a formal definition of History-Dependent Excitability (HDE) as a measure of the propensity to firing in any moment in time, linking the subthreshold history-dependent dynamics with spike generation. This definition allows the quantitative assessment of the intrinsic memory for different single-neuron dynamics and input statistics. We illustrate the concept of HDE by considering two general dynamical mechanisms: the passive behavior of an Integrate and Fire (IF) neuron, and the inductive behavior of a Generalized Integrate and Fire (GIF) neuron with subthreshold damped oscillations. This framework allows us to characterize the sensitivity of different model neurons to the detailed temporal structure of incoming stimuli. While a neuron with intrinsic oscillations discriminates equally well between input trains with the same or different frequency, a passive neuron discriminates better between inputs with different frequencies. This suggests that passive neurons are better suited to rate-based computation, while neurons with subthreshold oscillations are advantageous in a temporal coding scheme. We also address the influence of intrinsic properties in single-cell processing as a function of input statistics, and show that intrinsic oscillations enhance discrimination sensitivity at high input rates. Finally, we discuss how the recognition of these cell-specific discrimination properties might further our understanding of neuronal network computations and their relationships to the distribution and functional connectivity of different neuronal types.

## Introduction

Since the beginnings of electrophysiology it has been observed that neuronal firing rate in sensory systems carries information about the presented stimulus [1], [2]. Still, it has long been recognized that the exact timing of neural firing carries additional information beyond that provided by the mean firing rate (see, for example, [3]–[6]). In addition to electrophysiological studies based on a stimulus-response paradigm, precisely timed spiking patterns have also been observed in the spontaneous activity of a variety of preparations [7]–[9]. While these results might not apply universally in the nervous system [10]–[12], they are clearly advocating for an important role of precisely timed activity in neural network processing. The higher information capacity offered by precisely timed firing patterns is likely to be exploited by the nervous system, given that neurons are sensitive to the exact timing of applied stimuli [13], [14], and muscle contraction depends upon the temporal pattern of activity of its innervating motoneurons [15], [16]. Nevertheless the dynamical mechanisms underlying the sensitivity to temporally structured inputs are not completely understood.

Many biophysical mechanisms have been proposed as feasible candidates in decoding a temporal code. At the network level, a combination of delay lines (possibly tuned by spike timing-dependent plasticity) and an array of coincidence detectors allow the recognition of certain sequences of Inter-Spike Intervals (ISIs) [17], [18]. At the single-cell level, the interplay between short-term synaptic dynamics and intrinsic neuronal oscillations results in selective transmission of input bursts with a frequency content that matches the synaptic and intrinsic filtering properties [19].

When dealing with temporally selective neuronal mechanisms, most attention has been drawn on the role of short-term synaptic dynamics [20]–[22], based mostly upon mechanisms of vesicle depletion and replenishment which in turn depend upon several pre and post synaptic factors (for reviews see [23], [24]). In particular, it has been shown how the random but not independent synaptic transmission between subsequent synaptic event conveys information (in the formal sense of uncertainty reduction) about the timing of previous presynaptic spikes [25], [26].

The present study focuses on the other side of the engram [27], that is, on the history-dependent processing capabilities offered by intrinsic neuronal dynamics. These include ion channels activation - inactivation kinetics, intracellular second messenger processes involving calcium up-take and release from intracellular stores, and in general any neuronal process located within or across the cell membrane that could possibly alter its excitability or input-output mapping. The last decades have seen a renewed interest in intrinsic neuronal properties, and in particular in intrinsic oscillations [28], [29]. It has been shown that subthreshold intrinsic oscillations act as a neuronal band-pass filtering mechanism [31], [32] and shape the input-output relationship of single neurons [33]. For example, a certain neuron will respond to a train of synaptic events in a way that depends upon the precise temporal structure of the input train and upon the neuron's intrinsic properties. This study investigates more deeply into the relationship between intrinsic single-cell properties (in particular, intrinsic oscillations) and the encoding - decoding of a temporal code.

In any moment in time, a neuron carries information about its history of stimulation through its dynamical variables. In particular, the history-dependence of ion channel dynamics has long been recognized as a single-cell substrate for transient memory [34]–[38]. Certain specific ionic currents have been considered as molecular basis for single-cell memory, due to their slow dynamics of activation or inactivation, or the complex temporal profile of their responses [34], [37], [38]. The interplay between calcium influx (through synaptic and voltage-gated channels) and its diffusion and exchange through intracellular stores has also been suggested as a candidate mechanisms for single-cell memory [39], [40]. The positive feedback required for persistent activity can also be provided by a slow excitatory synapse of a neuron onto itself, i.e. an autapse [41]. The slow and non-linear dynamics of NMDA glutamate receptors, along with the impedance gradient resulting from dendritic tapering, have recently been proposed as a single-cell mechanism for the discrimination of spatiotemporal inputs [42]. However, the context-dependent computational capabilities of neurons might be better understood when all the intrinsic neuronal processes that influence its excitability are taken into account, and represented in a dynamical system framework. In the case of a biological neuron, the dynamical processes involved are very diverse in nature and span several time scales, from few milliseconds in the case of fast ion channels' activation-inactivation kinetics, until days or months in the case of neurite growth and protein synthesis. Computational models are extremely useful in taming the enormous complexity arising from such a picture, allowing the theoretical measurement of the discrimination capabilities of simple dynamical mechanisms which are general to several neuron classes.

In this paper we present a formal measure of the neuron's state directly related to spike generation, namely the History-Dependent Excitability (HDE), which lumps the different history-dependent dynamical variables of an arbitrary model neuron to a single, scalar value that describes its propensity to firing in any moment in time. This dynamical mnemonic trace allows the interpretation of newly incoming information in the context of the previously received inputs, and hence the discrimination of information at the single-cell level. The comparison between the HDE trajectories arising from different input histories quantifies the intrinsic discrimination capabilities of single neurons, and their dependence upon the input statistics and the neuronal dynamics.

Since different physiological processes can yield similar dynamical effects, it is useful to consider general, dynamical neuronal models which abstract from the specific molecular mechanisms involved. In particular, we illustrate the concept of HDE with two very general dynamical mechanisms: the RC behavior of a passive membrane, captured by a simple Integrate and Fire model, and the inductive RLC behavior of a neuron with a resonant current, described in its simplest (linear) form by a Generalized Integrate and Fire model (see Methods). These models are based on analogies with linear electric circuits, a formalism with a long and successful history in the phenomenological characterization of neuronal dynamics (for some early examples, see [43]–[46]). Before particularizing our analysis to these two simple cases, we will present a general mathematical framework that allows the quantitative assessment of the history-dependent modulation of HDE for arbitrary neuron models.

## Results

### History-Dependent Excitability (HDE)

In the most general mathematical framework a neuron is described as a dynamical system:
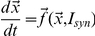



where 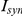 is the sum of all synaptic currents flowing through the neuron's membrane. Every synaptic event will move the current phase point according to the intrinsic and synaptic dynamics, while in between synaptic events, the phase point will evolve according to the intrinsic dynamics only. Since the outcome of a synaptic event depends upon the intrinsic state vector 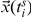 at the time of the event, and 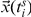 depends upon the state at the time of the previous synaptic event 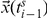 and the time between events 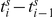, the neuron acts as a dynamic encoder of its stimulus history. That is, the neuron state vector will reflect the previous history of stimulation, and will itself determine the following evolution of the neuron as a dynamical system. This property is particularly suited for the encoding of precisely timed stimuli. In this article we analyze how the specific form of the system vector field 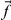 will determine the computational characteristics of the dynamic encoding mechanism, such as its efficiency in the presence of noise and its sensitivity to particular features of the input.

The neuron's state vector 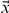 encodes stimulus history in an implicit way: the actual values of the dynamical variables are unknown, both to the experimenter and to other neurons in the network. Their values can only be indirectly inferred through their influence on membrane potential or intracellular calcium (the main physiological quantities directly measurable though standard experimental techniques) and, in particular, on spiking activity.

On the other hand the state vector 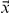, containing all relevant information about the neuron's dynamic state, directly affects the way the neuron responds to incoming stimuli. From the simplest point of view, input-output history modulates the neuron's excitability, or propensity to firing, as a function of time. More generally, input-output history can affect single-cell processing by varying the output repertoire a neuron can exhibit, for instance by augmenting the probability of short burst emission versus single spike emission in a certain time window. This phenomenon allows for history-dependent information processing: the neuronal input-output transformation is not a static map, but it is changing in time in a history-dependent manner. The influence of the past history on the current input-output transformation can be considered as a cell-based substrate for contextualization of information in the nervous system, or short-term memory [35].

Depending on the local context that the neuron ‘sees’ through the synapses impinging on it in the immediate past, it will differently treat the information it receives. As we will show in this paper, this context-dependent processing is a very general property of dynamical systems and it can be presented in its simplest form with linear models such as the IF and GIF neuron models.

The state vector contains all relevant information about the dynamic state of the neuron, hence it can be used to predict the neuron's response to an arbitrary stimulus. Nevertheless, it is not easy to deal with directly. In a model neuron the state vector's dimensionality can be high, and obviously it depends upon the specific model considered, making the comparison between different neuron models difficult and somewhat arbitrary. In a real neuron the state vector depends upon the description level one wishes to adopt, and even when just a few channels' activation-inactivation kinetics are taken into account, it is impossible to measure experimentally. Hence, it is useful to introduce a lumped, functional and physiologically motivated measure of the neuron's state, directly related to spike generation. To this end we propose a formal definition of History-Dependent Excitability (HDE) at any moment in time as the minimal synaptic strength 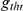 of an excitatory synaptic event that can make the neuron fire. This measure is obviously dependent upon the synaptic kinetics considered. In this work our focus is on intrinsic neuronal dynamics, hence we decided to adopt the simpler synaptic description: Excitatory Post-Synaptic Potentials (EPSPs) will be modelled as instantaneous, voltage-independent shifts in the 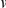 variable, representing the membrane potential. Numerical simulations with more realistic models, i.e. conductance-based model neurons with exponentially decaying synaptic conductances, yield similar results. As we show in this work, the time evolution of HDE 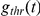 carries information not only about the intrinsic properties of the cell, but also about its input-output history.

### Example of single-neuron discriminability in minimal models

Traditionally the detection capabilities of neurons have been assessed by observing how their output changes as a function of their input. For example, two stimuli are considered to be indistinguishable as long as they both result in a single postsynaptic spike, or in the lack of it. We show that this is not necessarily the case: two different input stimuli can still be distinguished if they bring the neuron to a different internal state, and hence change its response properties to future stimuli.

**Figure 1:**
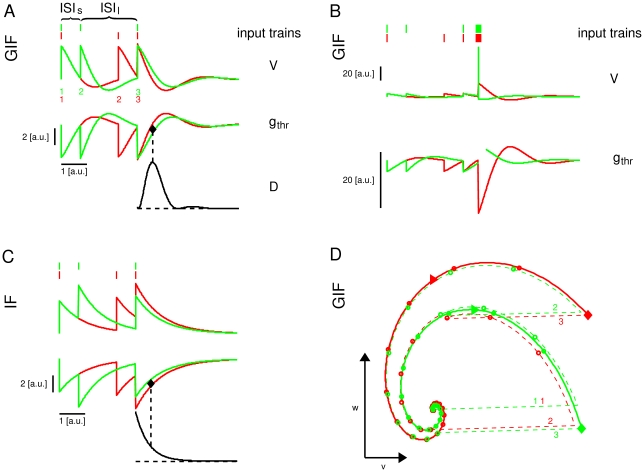
Illustration of the History-Dependent Excitability concept. Response of the GIF (A,B,D) and IF (C) model neurons to two different input trains, composed of the same ISIs. A: Voltage (top) and HDE (middle) trajectories arising from two different input trains (on top of the voltage traces). The instantaneous discriminability after the last spike of the train (black line) first increases and then decreases, resulting in an optimal time for discrimination based on HDE (black diamond): a presynaptic spike with the strength and the timing indicated by the black diamond will be suprathreshold after the red input triplet, but subthreshold after the green input triplet. B: As in A, but with the addition of a fourth presynaptic spike with the timing and amplitude indicated by the black diamond in A. The intrinsic discriminability is not shown. C: The same as in A, for the IF model neuron. For a purely passive neuron, the intrinsic discriminability is an exponentially decreasing function of time. D: Trajectories in the phase plane 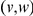 corresponding to the two input triplets in panel A. The trajectories are shown in dashed line before the last spike of the triplet, and in solid line after it. Circles are drawn every 0.25 u.t. A rightward triangle is drawn at the time of maximal discriminability (corresponding to the black diamond in A). Digits in A, D indicate the ordinal number of EPSPs in the train.

In the case depicted in Fig. 1, a GIF neuron with subthreshold damped oscillations (see Methods) receives one of two different input trains, comprising the same constituent ISIs, one long ISI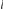 and one short ISI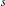 (Fig. 1A). Both input trains evoke subthreshold responses only, hence no differential information about the input history is delivered to other cells in the network. Importantly though, the information about which of the two input trains has been received is contained in the state variables immediately after the last spike of the train, and can be extracted from the subsequent free evolution of the state vector. The voltage 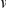 variable after the last spike of the train is almost identical for both inputs, because the internal variable 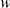 counteracts voltage changes, while in the IF neuron the input with increasing frequency evokes a greater depolarization (Fig. 1C). Nevertheless, the different histories result in different values of 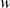, which in turn result in a history-dependent free evolution to the rest state (Fig. 1D): the 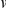 variable decreases smoothly and with a shallow, late sag after the (ISI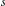,ISI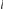) triplet, while it decreases more abruptly and with a deeper, earlier sag after the (ISI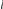,ISI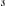) triplet. The black line in Fig. 1A shows the correspondent evolution of the instantaneous intrinsic discriminability (a measure of the difference in the HDE trajectories, see next section for a formal definition), which increases and then decreases, showing a maximum at a certain time after the last input spike.

This history-dependent free evolution of the model neuron allows for spike-mediated discrimination between the two different histories by a synaptic event with proper time and amplitude: a presynaptic spike with a time and an amplitude indicated by the black filled diamond in Fig. 1A, upper panel, results in a postsynaptic spike after history (ISI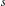,ISI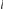), but only produces a subthreshold oscillation after history (ISI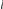,ISI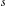) (Fig. 1B). In this example the stimulus time has been chosen at the time of the maximal instantaneous discriminability between the two input histories, and its amplitude as the middle point between the two HDE 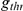 at that time. However, any input that lies in the space between the two different HDE trajectories, corresponding to the two different input histories, can in principle (disregarding the effects of noise) discriminate between the two cases. This does not mean than a single neuron actually discriminates between these inputs, but that it *could* discriminate between them based on the intrinsic memory brought about by its HDE trajectory. The actual binary discrimination is only realized if some specific inputs are received.

If the same input trains are delivered to an IF neuron with the same membrane time constant, they are still distinguishable but in this case the absolute difference between the two HDE trajectories decreases exponentially after the last spike of the train (Fig. 1C). Conversely, in the GIF model it first increases and then decreases, resulting in an optimal time for intrinsic discriminability. Note that the time of maximal intrinsic discriminability does not correspond in general to the time of maximal probability of spike emission. In the case shown in Fig. 1A, the time of maximal probability of spike emission is at the end of the input train, and actually corresponds to a near zero discriminability. Conversely, the time of maximal discriminability is when the range of near-threshold inputs which are suprathreshold after history 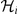, but subthreshold after history 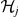, is maximal.

For these linear models, we are able to derive analytical expressions for the discriminability between two input histories 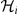 and 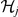, which we will use extensively in the rest of the paper.

### Instantaneous and cumulative discriminability

Living neurons *in vivo* typically receive synaptic signals from thousands of presynaptic cells. It seems that the synaptic strengths of the inputs converging to a given neuron are not exponentially distributed, but might be better described by a log-normal distribution [47]: this corresponds to the presence of a few, strong inputs that emerge from a multitude of much weaker connections [48], [49]. The last observation, along with the fact that neurons are extremely sensitive to correlations in their inputs [50], [51], led us to our operational approximation.

In this study, we consider the linearization of the neuron's dynamics around its “working point”, determined by its intrinsic properties and by the collective balance of the weak and asynchronous synaptic events it receives. The synaptic events will be considered as instantaneous, voltage-independent shifts in the 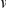 variable. These can represent the synchronous arrival of many weak EPSPs, as in thalamocortical connections [52], retinal projections to the thalamus [3], or projections from the antennal lobe to the mushroom body [5]. Equivalently, this formalism can also represent the synchronous arrival of a few strong EPSPs, or even a single, very strong EPSP. Hence, we consider the synaptic kinetics to be much faster than the membrane time constant, or the resonant current activation rate (in the GIF model).

This approximation allows us to calculate the neuron's response to a train of presynaptic events with an iterative formula, and to calculate the discriminability based on intrinsic properties analytically (see Text S1). The discriminability between two input histories aims at measuring the difference in the corresponding evolutions of the HDE. The most natural definition is then the square difference between the two HDE trajectories. In mathematical terms, we define the instantaneous discriminability between the input histories 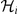 and 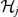, resulting in the phase points 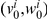 and 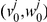 at time zero (which we set for convenience immediately after the last spike time of the input train), as
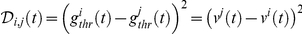



where the last equality is due to the fact that in these linear models the firing threshold depends upon the voltage variable 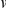 only. Substituting the analytical solutions in the case of the IF and GIF models (see Text S1 for details) one obtains:
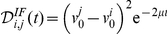


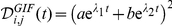



where 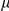 is the leak conductance in the IF model; 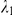 and 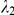 are the eigenvalues of the GIF model, and 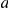 and 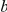 are coefficients that depend upon the model parameters and upon the difference in initial conditions 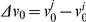 and 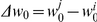.

In the case of an IF neuron the instantaneous discriminability is an exponentially decreasing function of time, meaning that the intrinsic memory about the previous history will quickly vanish, and practically be lost after a few membrane time constants. In the case of a GIF model with real eigenvalues, the picture does not differ much, with the exception that now the exponential decay is governed by more than one time constant. In the case of a GIF model with intrinsic oscillations, that is a GIF model with complex conjugate eigenvalues 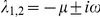, the instantaneous discriminability can be written more conveniently as
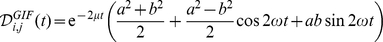



In this case, the instantaneous discriminability is an exponential decreasing function of time multiplied by a sinusoid. This means that for certain input histories 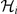 and 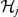, the maximal instantaneous discriminability can be achieved for times greater than zero, as in the example shown in Fig. 1.

The instantaneous discriminability is a measure of the square difference between two HDE trajectories originating from two input histories 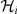 and 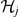 in any moment in time. Its integral in time from zero to 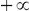, which we call cumulative discriminability, is a measure of the information about the discriminability between the two histories 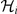 and 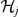 that could be gathered by observing the instantaneous discriminability for an infinite amount of time. This measure can be geometrically interpreted as the square area between any two HDE trajectories like those depicted in Fig. 1.
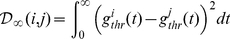
(1)


After some analytical calculations (see Text S1 for details) we get
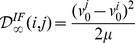
(2)

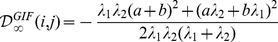
(3)


If 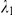 and 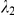 are complex conjugate we can write the last expression as
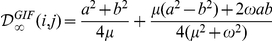



For both models the cumulative discriminability is a quadratic function of the difference in the state variables at time zero.

It can now be appreciated that the discriminability between two input histories 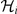 and 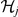 depends on the one hand upon the free evolution from the difference in initial conditions 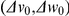, and on the other hand upon the encoding properties of the model neuron, that is, upon how the phase point 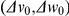 depends upon the input histories.

In our analysis we considered two different measures of the intrinsic discriminability: the cumulative discriminability and the maximal instantaneous discriminability. We did not find important differences between these measures, hence we present our results using the cumulative discriminability (which we will denote simply as 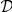) for its analytical simplicity.

### Discriminability between pairs of input trains

If the input history to a neuron can be considered as a sum of stereotypical synaptic potentials, discriminability between pairs of input trains can be computed analytically in a straightforward manner for the linear models considered in this study. If input history 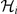 is composed of synaptic potentials evoked at times 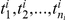, with 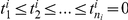 (without loss of generality we set the time reference at the last spike of the input train), the intrinsic discriminability between 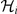 and 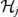 can be written as
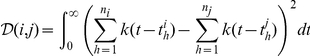



where 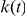 is the PSP kernel, reflecting the interplay between intrinsic and synaptic properties. Hence, the only input spikes which play a role in the intrinsic discriminability are those that do not occur simultaneously in the input histories 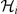 and 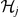. An instructive example is when the two input trains are equal in the number of spikes they are made of (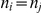), and in the spike times of each of their constituent events, except one. In this case, the previous expression reduces to
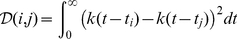
(4)


where we reindexed the spike times for simplicity. In this work our focus is on intrinsic neuronal dynamics, hence we describe synaptic potentials as instantaneous shifts in the voltage variable. In this case the PSP kernels for the IF and GIF neurons are (see Text S1 for details)
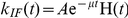


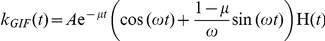



with 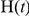 being the Heaviside functions, ensuring causality. Substituting the above expressions in (4) yields:
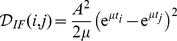
(5)

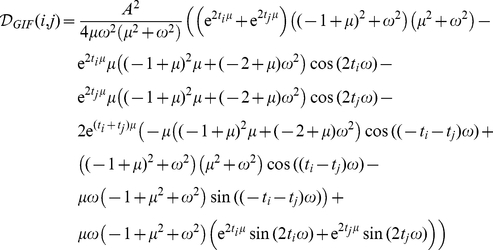
(6)


Since we are considering linear neuron models, these results also hold if the inputs are composed of the sum of the input trains considered above, plus any term which is equal among different input histories. This situation comprises a number of illustrative cases, which we detail below.

### Intrinsic discriminability between input pairs

Let input history 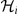 be composed of a pair of synaptic potentials evoked at times 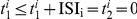, and input history 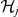 at times 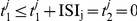. In this case the intrinsic discriminability is given by expressions (5) and (6), substituting 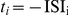, 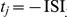.

### Intrinsic discriminability between input triplets with the same total duration

Let input history 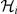 be composed of a triplet of synaptic potentials evoked at times 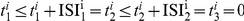, and input history 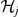 at times 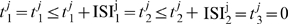. In this case the intrinsic discriminability is given by expressions (5) and (6), substituting 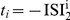, 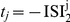.

### Intrinsic discriminability between input trains that only differ in the their second spike time

Let input history 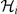 be composed of a train of synaptic potentials evoked at times 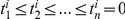, and input history 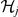 at times 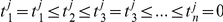, with 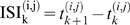 for 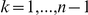. Let 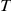 be the total duration of the input trains: 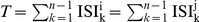. In this case the intrinsic discriminability is given by expressions (5) and (6), substituting 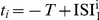, 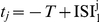:
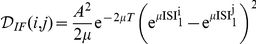


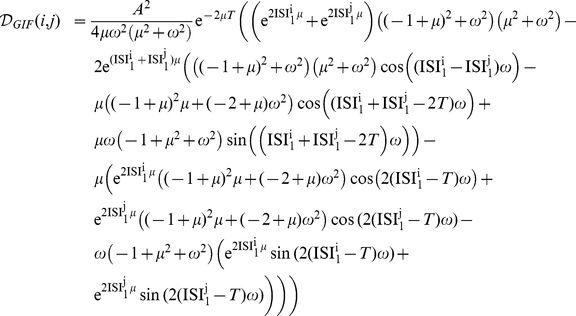



Hence, in these linear models the intrinsic memory about the input ISIs received further back in the past decreases exponentially with time.

**Figure 2:**
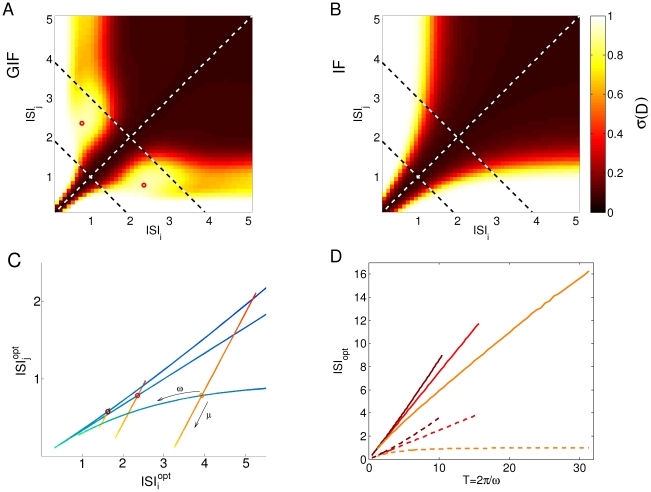
History-Dependent Excitability discriminates between input doublets. A, B: Intrinsic discriminability between input doublets ISI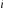 and ISI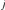 for the GIF (A) and IF (B) model neurons. Intrinsic oscillations enhance discriminability for input ISIs slightly shorter than the intrinsic period 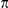. Local maxima of 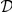 are indicated by red circles. Dashed lines indicate the bisectrix ISI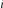 = ISI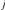, and the lines of constant mean ISI 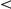
ISI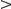 = 1 and 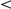
ISI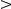 = 2, along which 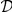 is plotted in Fig. 3B. 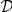 values have been passed through the sigmoidal function 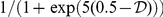 to improve visualization. C: Local maximum of 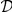 as 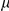 or 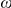 are varied, one at a time, between 20 and 500% of their starting values. Shades from yellow to red indicate decreasing values of 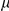, shades from green to blue indicate decreasing values of 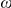. Local maxima corresponding to starting values for the parameters are indicated with circles. Brown Circle, 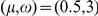; Red Circle, 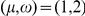; Orange Circle, 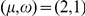. D: ISI corresponding to the local maximum of 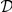 as 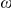 is varied. If (ISI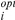,ISI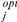) is the position of the local maximum and ISI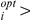 ISI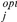, ISI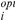 is drawn with a solid line, ISI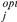 with a dashed line. Color code as in C.

Figure 2 shows expressions (6) and (5) for our canonical GIF and IF neurons (see Methods), as functions of ISI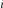 = 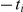 and ISI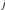 = 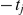. It can be seen that the discriminability decreases for longer and more similar input ISIs in both models. While this decrease is non-specific for the IF neuron, a more complex structure is observed in the GIF neuron. Indeed, a local maximum is observed for the GIF model at a certain value of (ISI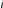,ISI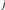).

The position of the local maximum (ISI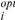,ISI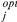) as a function of 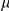 and 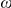 cannot be obtained analytically. We performed some numerical explorations varying 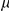 or 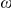, one at a time, between 20 and 500% of their initial values, starting from a few representative points in the (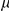,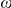) plane (Fig. 2C). Our results show that 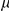 and 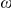 interact in a non-trivial way in determining the location of the local maximum in the (ISI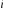,ISI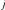) plane. In general, increasing 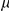 shifts the position of the maximum towards the closer axis, in a direction which doesn't depend much upon 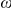. Decreasing the intrinsic frequency 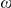 correspondingly shifts the maximum to lower input frequencies. This shift follows straight lines in the (ISI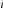,ISI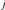) plane, but it bends towards the closer axis if 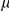 is high enough. This is consistent with the intuition that a neuron with a high effective membrane rate constant will poorly discriminate between low-frequency inputs. In particular, ISI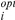 scales linearly with the intrinsic period of oscillations 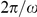 with a slope smaller than one, which decreases with increasing 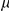 (Fig. 2D).

**Figure 3:**
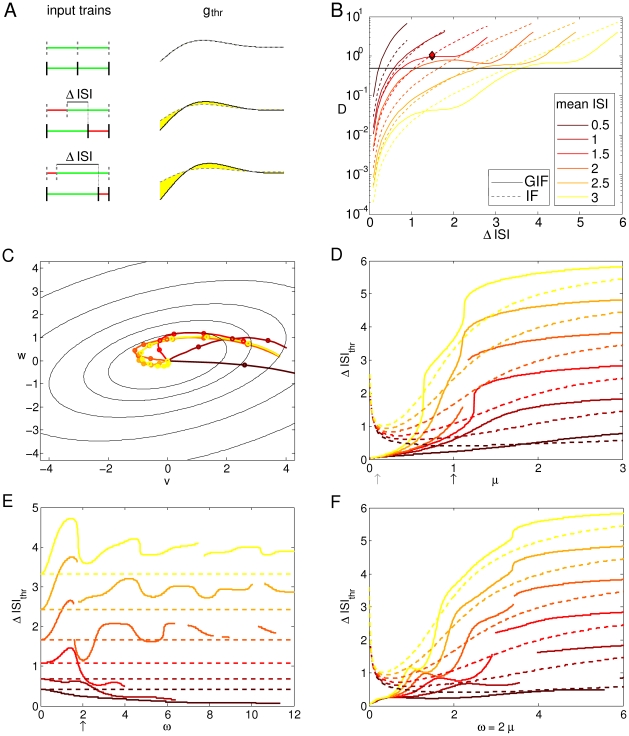
Sensitivity of intrinsic discriminability to temporally precise inputs. A: Input trains (left) and correspondent HDE trajectories after the last spike of the input train (right). As the input ISI difference 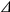
ISI increases, the resultant HDE trajectories diverge and the cumulative discriminability 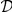 (yellow shaded area) increases. Note that the total duration of the input train is held constant. B: Intrinsic discriminability 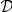 as a function of 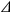
ISI for the GIF (solid line) and IF (dashed line) neurons. The general trend is an increase in 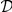 with increasing 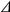
ISI, but an oscillatory component is superimposed on this trend in the GIF neuron. Shades from brown to orange indicate increasing mean ISI (see legend). The diamond indicates the pair of ISIs used in Fig. 1. The threshold of putative physiological significance 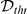 is depicted as a black horizontal line. C: Trajectories in the phase plane 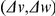 corresponding to the solid curves in B. Lines of constant discriminability are depicted as black ellipsis. D: Minimal ISI difference 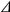
ISI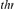 corresponding to an intrinsic discriminability above a threshold 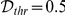 as a function of 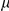, for fixed 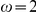. Colors and line styles as in B. E: 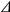
ISI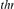 as a function of 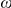, for fixed 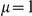. F: 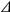
ISI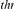 as a function of 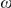, scaling 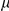 in order to maintain a fixed 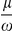 ratio of 0.5. Black arrows in D and E indicate the default parameter set; the correspondent curve of discriminability vs. 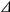
ISI is depicted in panel B. The gray arrow in D indicates the value of 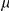 used in Fig. 4.

The values of the discriminability on the bisectrix ISI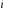 = ISI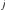 is zero, since identical input trains are not distinguishable. The evaluation of the intrinsic discriminability (6) and (5) along lines departing orthogonally from the bisectrix is especially interesting, since it denotes the discriminability between pairs of input trains with the same total duration, as the second-to-last spike is slightly advanced or delayed (see Fig. 3A).

The evaluation of (6) and (5) along (ISI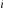 = 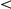
ISI
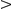
+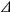
ISI/2, ISI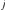 = 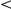
ISI
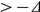
ISI/2) for different values of the mean ISI is plotted in Fig. 3B. When the average input frequency is high (with respect to the frequency of intrinsic oscillations), the discriminability increases rapidly with 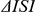 in both models, but the slope is higher for the GIF neuron (mean ISI
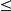
1). When the input ISIs are so short, the post-synaptic effects of subsequent spikes add almost linearly, and the dynamic encoding mechanisms provided by the neuron's intrinsic properties play little role. In this case, the difference in discriminability is mainly determined by the free evolution of the model neurons. In this regime, higher input frequencies evoke stronger depolarizations, which in turn result in free evolutions of greater amplitude and hence greater discriminability.

For input trains with frequencies close to the GIF intrinsic frequency, the discriminability increases faster for the GIF neuron, where it reaches a plateau or local maximum (Fig. 3B). After this plateau, discriminability will further increase only for very short ISIs, which corresponds to almost linear summation of the postsynaptic effects. For sufficiently long mean ISI (mean ISI
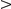
2) and long ISI difference, the intrinsic discriminability reaches a maximum value (independent of the mean ISI and of the ISI difference) corresponding to the difference in initial conditions corresponding to one and two EPSPs received at rest. Indeed, when one of the constituent ISI is greater than a few membrane time constants, the neuron has time to relax to its rest state almost completely and hence loses all the information about its previous stimuli.

In order to understand these observations one needs to take into account the contributions of two different effects: 1) the dynamic encoding of input history, which determines how subsequent presynaptic spikes affect the dynamic variables of the model in a history-dependent manner, and 2) the free evolution of the system after the last spike of the train (see also Fig. 7).

Both these ingredients are represented in Fig. 3C, where the trajectories in the phase plane 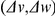 as the ISI difference is varied are represented together with the cumulative discriminability isolines, for several values of the ISI mean. We have shown before (expressions 2 and 3) that the intrinsic discriminability between two input histories 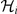 and 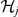 is a function of the difference in the dynamical variables after the last spike of the input train 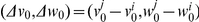. Hence, the dependence of 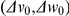 upon input frequency and ISI difference determines the dynamic encoding properties of the neuron. When the average input frequency is high, 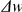 changes only slightly with the ISI difference, and the trajectories lie close to the 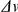 axis: in this regime the dynamic encoding mechanisms play little role. The greater the ISI difference, the greater 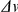 and hence the cumulative discriminability. When the average ISI is higher (mean ISI
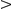
1.5) these trajectories acquire a curved shape, which indicates that the dynamical encoding mechanism is giving a significant contribution by modulating the slow variable 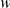 in a history-dependent manner: first the phase point leaves the origin in a direction that depends upon the mean ISI, and then moves tangentially to the discriminability isolines, which is consistent with the plateau in discriminability observed in Fig. 3B. It is worth noting that the plateau level does not depend upon the mean input frequency, but only on the synaptic strength and the neuron's intrinsic properties, and arises as soon as one of the constituent ISI is around half the intrinsic period of the model neuron.

These results suggest that the dynamical encoding mechanisms provided by intrinsic oscillations increase the sensitivity of short-long vs. long-short ISI discrimination. To clarify this point, we made the hypothesis that 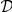 values above a certain threshold 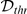 allow a reliable discrimination between pairs of input trains, in spite of the several non-deterministic phenomena observed in neurons (which we do not model explicitly). Hence, we defined the intrinsic sensitivity 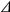
ISI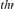 as the minimum ISI difference such that 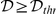. This measure assesses the sensitivity of the HDE in discriminating between “accelerating” and “decelerating” triplets with the same average frequency (Fig. 3D–F).

In the case of the IF neuron, 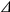
ISI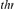 can be calculated analytically by inverting expression (5):
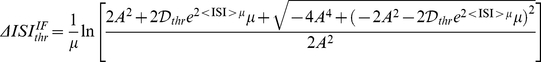



The corresponding expression (6) for the GIF neuron is not invertible, hence we set 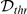 arbitrarily at 0.5 (black horizontal line in Fig. 3B) and calculated 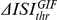 numerically. For the GIF neuron, discriminability curves as a function of 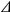
ISI are not always monotonic, and for specific parameter sets the discriminability curves crossed the threshold 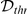 more than once. In these cases we did not define 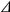
ISI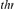 and they appear as blank spaces in Fig. 3D–F.

**Figure 4:**
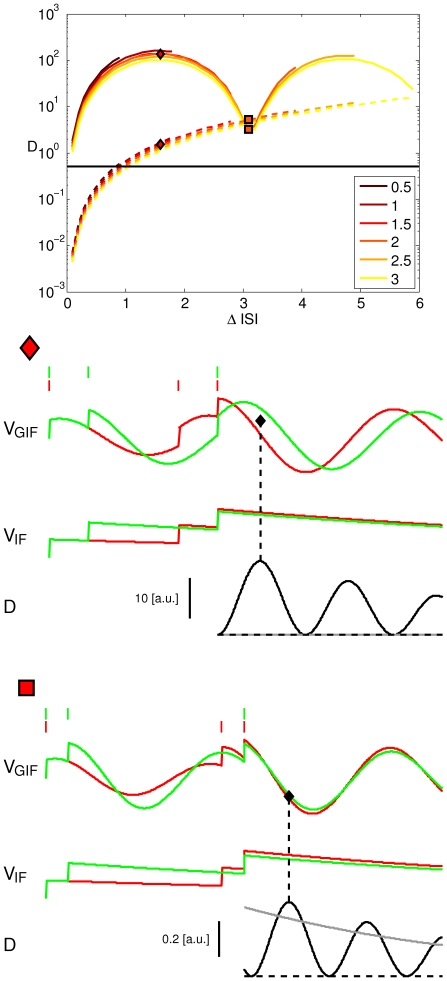
Intrinsic discriminability in the GIF and IF models for slow effective membrane rate constant 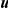. Top panel: Cumulative discriminability as a function of 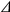
ISI for input triplets of fixed duration for the GIF (solid line) and IF (dashed line) neurons. Shades from brown to orange indicate increasing mean ISI (see legend). The threshold of putative physiological significance 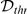 is depicted as a black horizontal line. The symbols (red diamonds and red squares) indicate the pairs of ISIs used in the lower panels, and the resulting cumulative discriminability. Lower panels: Voltage traces of the GIF (top) and IF (middle) neurons in response to two different input trains, composed of the same ISIs, and the evolution of their instantaneous discriminability (bottom; black line for the GIF model and gray line for the IF model) after the last spike of the input train. Note the different vertical scale for the HDE in each panel. The black diamond indicates the time of maximal instantaneous discriminability in the GIF neuron, which correspond to the time of greater separation in the voltage trajectories resulting from the two different input trains. Parameter set: 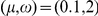. Note that, as the membrane rate constant 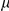 approaches zero, the IF neuron tends to reflect only the number of spikes received, regardless of their timing. In the same conditions, the GIF neuron will continue to oscillate indefinitely with a phase and amplitude that depend upon the precise timing of the input train.

Decreasing 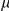 results in shorter 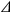
ISI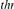, and hence greater sensitivity in the discrimination between different input trains (Fig. 3D). This is an expected result since 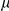 is the rate of decay of the model variables to the rest state, and determines for how long the information about previous stimuli will be available in the HDE trajectory. The sensitivity is better in the GIF neuron for 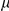 smaller than a certain value, which increases with increasing input frequency. While the sensitivity always improves with decreasing 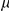 in the GIF neuron, it starts worsening at a certain, unrealistically low value of 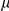 (which increases with increasing input frequency) in the IF neuron. Indeed, when the effective leak 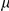 tends to zero, the IF neuron acts as a perfect integrator, and its membrane potential (and hence its HDE) comes to reflect solely the number of spikes received, while the precise timing of the received spikes becomes irrelevant. In this limit, 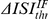 tends to infinity. In the same limit, the GIF neuron acts as an undamped oscillator, resulting in an infinitely high cumulative discriminability as soon as the input trains differ, and hence a vanishing small 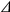
ISI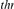 (Fig. 4). At high values of 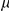, the post-synaptic effects decay very fast, and significant discriminability values are achieved only when one of the two ISIs is very small. Hence, 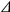
ISI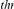 tends to the higher possible value of twice the mean ISI.

The effect of the intrinsic frequency 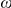 is somewhat more complicated (Fig. 3E). For high input frequencies, the sensitivity improves with increasing 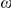, because of both the increased discriminability based on the free evolution and the greater encoding capabilities resulting from intrinsic oscillations (see also Fig. 7). When the intrinsic frequency 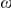 exceeds a certain threshold (which increases with increasing input frequency), the profile of the intrinsic discriminability as a function of the ISI difference becomes oscillatory even at high input rates and can result in very low values for specific input triplets, a phenomenon conceptually remindful of destructive interference in classical wave theory (see also Fig. 4). For lower input frequencies, the minimal detectable difference 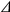
ISI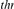 as a function of 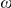 is oscillatory: it first increases until a certain value of 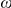 (which depends only weakly on the input frequency), then decreases, and then tends to stabilize through oscillations of progressively smaller amplitude and higher frequency. As soon as the input frequency is lower than a certain threshold, the neuron with intrinsic oscillations is less sensitive than the IF neuron for every value of 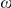, suggesting that intrinsic oscillations improve the intrinsic discriminability only for input trains with frequency close to, or higher than, the intrinsic frequency of oscillations.

Note that as we increase the membrane rate constant 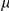 or the oscillation frequency 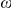, the damping coefficient 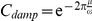 (defined as the ratio between the second and the first peak in the free evolution of the voltage variable from an initial condition different than rest) also varies, and consequently the oscillating character of the neuron.

In order to separate the effects due to 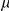, 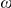 and to the damping coefficient 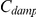, we performed some additional calculations keeping a constant 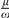 ratio of 0.5, as in the canonical GIF model. The results of this analysis are shown in Fig. 3F. Varying 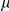 and 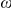 proportionally (thus maintaining a constant damping coefficient 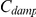) has similar effects on the sensitivity 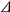
ISI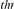 as varying 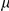 while keeping 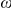 fixed: the GIF neuron exhibits a better sensitivity (smaller 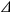
ISI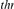) than the IF neuron for 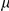 smaller than a certain threshold, which increases with increasing input frequency. When maintaining a constant damping coefficient 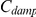, though, the increase in 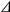
ISI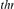 with increasing 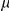 is smoother and the sensitivity curves are generally below the correspondent curves in Fig. 3D, where 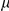 was varied while keeping 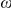 fixed. This suggests that the steep increase in 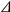
ISI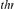 with increasing 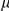 observed in Fig. 3D is also due to the decrease in the damping coefficient 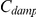, in addition to the decrease in sensitivity resulting from a faster convergence to the steady state.

The results exposed in this section can be directly applied to the characterization of biological neurons, and might be used to test the physiological relevance of the phenomenon for different cell types and in different brain areas. For instance, from standard electrophysiological measures such as a neuron's (complex) impedance, we could obtain estimated values for the neuron parameters 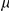 and 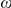 (see for example [32] for a fitting procedure). On the other hand, the variance of the membrane voltage due to random fluctuations could be directly related to a value of 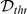, above which discriminability values are expected to be efficiently exploitable for contextualized information processing. Such analysis could assess the robustness of the intrinsic discriminability as a function of the input frequency, or the amount of coherence in the presynaptic population needed to form a signal that could be efficiently encoded through the intrinsic dynamical mechanism described.

### Intrinsic discriminability between random input trains

In the previous section, we analyzed the input discrimination capabilities based on intrinsic single-cell dynamics between some particular, well defined inputs. In this section, we wish to generalize the previous analysis and characterize the intrinsic discriminability as a function of input statistics and intrinsic properties for random input trains. In particular we considered input trains composed of exponentially distributed ISIs. This distribution is particularly significant in neuroscience, since neurons in many brain regions exhibit a firing statistics that is well fitted by an exponential distribution with a refractory period [53].

The average discriminability between pairs of input trains that only differ in their second to last spike can be easily calculated by integrating (4) over the exponential probability density:
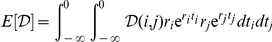



where 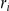 is the mean firing rate of input history 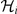. Solving yields:
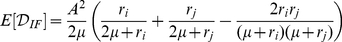
(7)

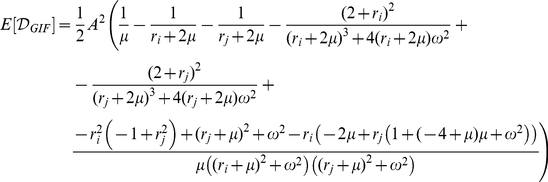
(8)


**Figure 5:**
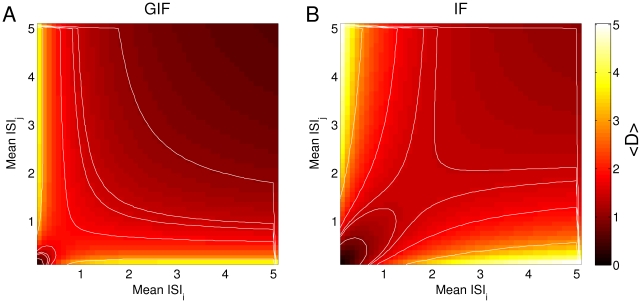
Average discriminability between random input doublets. Discriminability between input doublets with ISI extracted from an exponential distribution with mean values ISI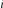 and ISI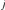, averaged over 10000 pairs. Isolines are drawn for 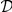 values of 1, 1.4, 1.5, 1.8, 2.8. In the range of input ISIs which interact with the intrinsic neuronal dynamics, discriminability is higher between input doublets with different frequencies than between doublets with the same frequency in the IF neuron. This is not observed in the GIF neuron, where the discriminability is mainly determined by the frequency of the faster input.

Expressions (8) and (7) are shown in Fig. 5 as a function of 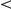
ISI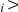 = 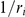 and 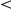
ISI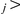 = 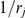 for our canonical GIF and IF neurons (see Methods). For the GIF neuron the average discriminability between random input doublets with the same average frequency is almost as good as between random input doublets with different average frequencies, and seems to depend mainly upon the frequency of the faster input. Indeed, isolines of constant discriminability are orthogonal to the bisectrix 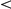
ISI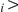 = 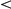
ISI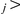, and then run parallel to the main axes (Fig. 5A). This behavior corresponds to sensitivity to input ISIs rather than average rates, given that input doublets with different rates are not better discriminated than input doublets with the same rate. Conversely, the IF neuron shows a greater sensitivity to input rate: pairs of input doublets with the same average rate yield lower discriminability values than pairs of input doublets with different average rate. In fact, for a purely passive neuron, the isolines of constant discriminability are approximately parallel to the bisectrix 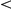
ISI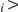 = 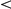
ISI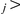 (Fig. 5B). Rate sensitivity decays for low frequency inputs, for which the average discriminability is mainly determined by the frequency of the faster input. In this regime the GIF and IF neurons behave similarly.

**Figure 6:**
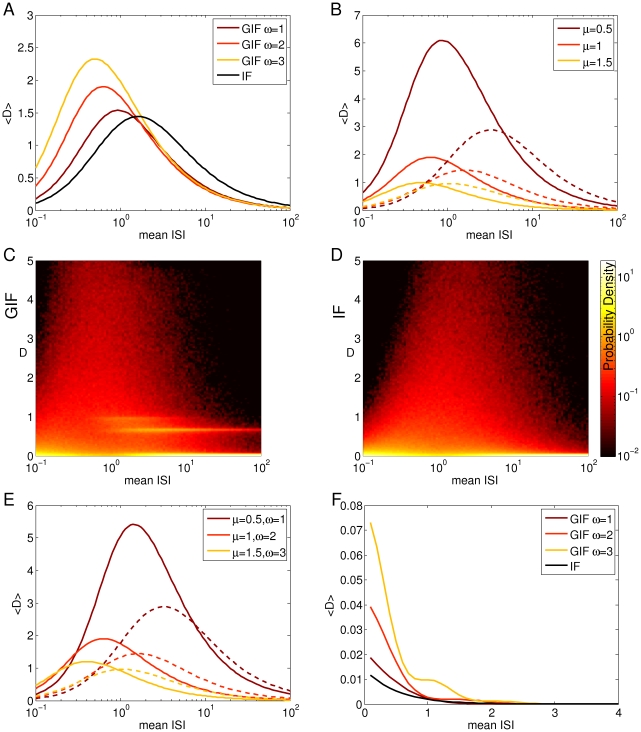
Intrinsic discriminability as a function of input statistics for different model neurons. A: Mean discriminability between input triplets with exponentially distributed ISIs, as a function of the input mean ISI, for the GIF and the IF neuron. Shades from brown to orange indicate GIF neurons with increasing values of the intrinsic frequency 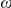 (see legend), while the black line indicates the IF neuron. The membrane rate constant 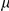 was fixed at its canonical value of 1. B: The same as in A, but 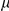 has been varied while 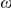 was fixed at its canonical value of 2. Shades from brown to orange indicate increasing values of 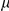 (see legend). GIF neurons, solid lines; IF neurons, dashed lines. C, D: Probability densities of the discriminability between input triplets with exponentially distributed ISIs, as a function of the input mean ISI, for the GIF (C) and the IF (D) neuron. E: The same as in A and B, but 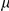 and 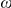 have been scaled proportionally in order to maintain a fixed 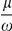 ratio of 0.5. Lighter colors indicate greater values of 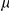 and 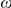. F: The same as in A, for gaussianly distributed input ISIs. Summarizing, increasing 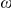 increases the discriminability at high input rates, while decreasing 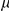 increases the discriminability especially for middle and low frequency inputs.

Evaluation of (7) and (8) along 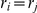 returns the average discriminability between input trains extracted from the same exponential distribution, which we plot as a function of the mean ISI in a semilog scale in Fig. 6A. The black line indicates the IF model, while lighter colors refers to GIF models with increasing values of the intrinsic frequency 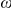 (see caption). For both models, the discriminability first increases and then decreases, showing a maximum at an intermediate value of the mean ISI. For the exponential distribution, the mean equals the standard deviation: hence spike triplets with short ISIs are poorly discriminated because they are composed of very similar ISIs. Indeed, analogous calculations using gaussianly distributed ISIs (see Text S1) with a very narrow (and constant) standard deviation shows a decreasing trend for increasing mean input ISI (Fig. 6F), indicating that the increasing trend at high input frequencies depends upon the standard deviation of the input. The discriminability for long ISIs is poor too: for such distributions most of the ISIs are longer than the time span of HDE, the neuron relaxes almost completely to its rest state and the dynamic trace reflecting its previous stimuli is lost. The maximum is achieved when the variability is great enough, yet the mean ISI is not longer than the time span of intrinsic memory.

**Figure 7:**
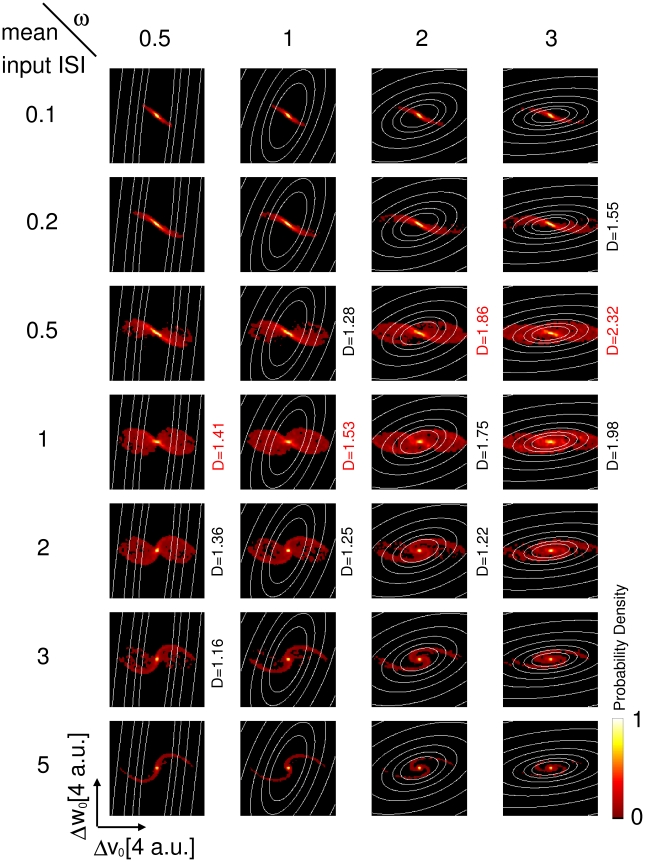
Intrinsic discriminability is determined by the interplay of the dynamic encoding and the free evolution. Probability distributions of the difference in the dynamical variables immediately after the last spike of the input train 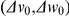 for different values of the average input ISI, which increases along the rows (0.1, 0.2, 0.5, 1, 2, 3, 5). The isolines of the cumulative discriminability as a function of 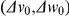 are represented as white lines. Isolines are drawn for discriminability values of 1, 2, 4, 8, 32 and 64, starting from the center. Probability densities are normalized to the peak value in each plot. For each value of 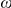, which increases along columns, the three higher 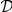 values are shown (the higher 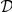 value is shown in red). For high frequency inputs, the dynamic encoding mechanism plays little role, and the 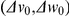 points accumulate along a line. As the input frequency decreases, the input trains interact with the intrinsic oscillatory dynamics and the 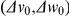 points distribute over a larger area in the 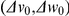 phase space. Note that the input mean ISI that results in the greatest spread of points in the 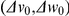 phase space, which corresponds to the optimal input frequency for dynamic encoding, decreases with increasing 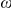. In addition to this, increasing 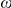 results in a greater average discriminability for 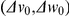 points in a circular region centered at the origin, denoting a greater discriminability based on the free evolution of the model neuron.

As the intrinsic frequency 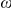 increases, the intrinsic discriminability increases almost linearly for short input ISIs, and the peak of maximal discriminability shifts to higher input frequencies. This effect is due to two different mechanisms: on the one hand an increase in 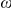 allows the intervention of the dynamical encoding mechanisms at higher input frequencies, and on the other hand it allows a better discriminability based on the free evolution of the model neuron (Fig. 7). The former feature is reflected in the distribution of 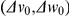 points, and the latter in the 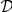 isolines, depicted in the panels of Fig. 7. As noted previously (expressions 2 and 3), the intrinsic discriminability between two input histories 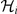 and 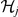 is a function of the difference in the dynamical variables after the last spike of the input train 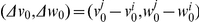. Hence, the distribution of 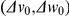 points is an indicator of the dynamic encoding properties of the neuron, that is, of how the input statistics is transformed into a distribution of internal states through the neuron intrinsic dynamics. Figure 7 shows the probability densities of 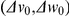 for different input statistics and neuron parameters. For high frequency inputs, the dynamic encoding mechanism plays little role, and the 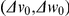 points accumulate along a line. As the input frequency decreases, the input trains interact with the intrinsic oscillatory dynamics and the 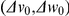 points distribute over a larger area in the 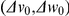 phase space. As the input frequency decreases further, most input pairs result in 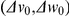 values close to the origin. Indeed, if the input ISIs are long compared with the membrane time constant, the phase point relaxes almost completely to the rest state in between synaptic events and the intrinsic memory about previous inputs is lost. In this low frequency input regime, the intrinsic discriminability decreases slightly for increasing intrinsic frequencies, but saturates at approximately 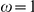, so that an additional increase in the intrinsic frequency does not affect discriminability (Fig. 6A). Note that the input mean ISI that results in the greatest spread of points in the 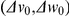 phase space, which corresponds to the optimal input frequency for dynamic encoding, decreases with increasing 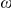. This is consistent with the shift to higher input frequencies of the peak in the discriminability as 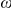 is increased (Fig. 6A). In addition to this, increasing 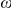 results in a greater average discriminability for 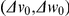 points in a circular region centered at the origin, denoting a greater discriminability based on the free evolution of the model neuron. Again, note that a higher discriminability at high input rates in the GIF neuron does not correspond to a higher probability of spike emission. Indeed, a purely passive neuron will be more likely to generate action potentials in this regime, because intrinsic oscillations counteract voltage changes and tend to keep the voltage in a narrow range around the resting potential. Conversely, a higher value for the average discriminability in the GIF neuron means that in this regime input trains will yield a more different evolution of History-Dependent Excitability, and will hence result in more different suprathreshold responses when the neuron is probed with near-threshold inputs.

Increasing 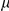 results in an overall decrease of the cumulative discriminability, which is more pronounced at low input frequencies. The discriminability peak slightly shifts to higher input frequencies (Fig. 6B). As in the sensitivity analysis carried out in the previous section, this is an expected effect since 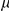 is the rate of decay of the model variables to the rest state, so that greater values of 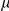 result in faster forgetting of the previously received stimuli. This effect is more pronounced at low input rates, because the model neuron has time to converge almost completely to its rest state in between synaptic events.

The standard deviation of the mean discriminability values depicted in Fig. 6A,B is almost equal to the mean (not shown). Indeed, a closer inspection of the distribution of discriminability values (estimated numerically, see Methods) reveals that most ISI pairs yield very small discriminability values, while a certain subset clusters around a discriminability value that corresponds to the plateau level in Fig. 3B (Fig. 6C). This phenomenon is exclusive to the GIF neuron and it is not observed in the IF neuron, where the distribution shows a peak at zero and a smooth decline for higher values of the discriminability (Fig. 6D). We interpret this phenomenon as a form of modulation of the intrinsic discriminability due to intrinsic oscillations: intrinsic oscillatory properties push the discriminability values between certain input train pairs above a certain threshold. If the threshold corresponded to a physiological limit of intrinsic discriminability, determined by the stochastic properties of neurons, this phenomenon would increase the sensitivity of the intrinsic discriminability between “accelerating” and “decelerating” input trains. This is a direct consequence of the steep rise to a plateau in the discriminability as a function of the ISI difference depicted in Fig. 3B.

Note that as we increase the membrane rate constant 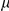 or the intrinsic frequency 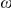, the damping coefficient 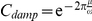 (defined as the ratio between the second and the first peak in the free evolution of the voltage variable from an initial condition different than rest) also varies, and consequently the oscillatory character of the neuron. In order to separate the effects due to 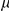, 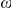 and to the damping coefficient 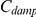, we performed some additional simulations keeping the damping coefficient fixed, and ranging 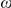 and 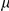 accordingly. The results of this set of simulations are depicted in Fig. 6E, where the membrane rate constant 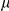 and the intrinsic frequency 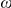 has been changed proportionally, maintaining a constant 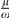 ratio of 0.5. Increasing the intrinsic frequency 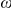 and the membrane rate constant 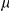 in a fixed ratio induces a slight increase in discriminability for high frequency inputs (due to the increase in 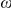), but also a substantial decrease in discriminability for low input frequencies (due to the increase in 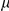). Also, it shifts the peak in discriminability to higher frequencies. The value of the peak decreases substantially when the cumulative discriminability is considered, but when the maximal discriminability is taken into account the decrease is minimal.

Taken together, these results can be summarized as follows: 1. Increasing the intrinsic frequency 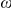 increases the discriminability between high frequency inputs. This happens because a fast 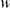 variable has time to activate in between synaptic events even at high input rates, enabling a dynamic encoding of input histories. In addition to this a fast intrinsic frequency increases the discriminability based on the free evolution of the neuron model. 2. Decreasing the membrane rate constant 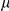 increases the discriminability especially for middle and low frequency inputs, because of the widening of the integration window of the neuron, and because the slower exponential decay of the instantaneous discriminability results in a stronger and longer-lasting memory of the immediate past.

## Discussion

Neurons are dynamical entities which act on several time scales; this endows them naturally with a capacity for short-term memory or context-dependent processing. The history of inputs to a neuron is dynamically encoded in its state-dependent, time-varying properties: each synaptic event induces some modifications in the neuron dynamical state which depend upon the previously received stimuli and their timing. In order to map intrinsic neuronal state to a physiologically, spike-related measure, we introduced the concept of History-Dependent Excitability (HDE), which we define as the minimal strength of a synaptic event that can cause the neuron to fire. As the neuron's dynamics unfolds in a history-dependent manner, the time evolution of the HDE carries information about the history of stimulation in the immediate past, allowing for context-dependent signal processing. This framework allows us to compare the context-dependent processing capabilities of different neuron types.

### History-dependent discriminability for different subthreshold dynamics

For the sake of illustration we considered linear models with a fixed voltage threshold for spike generation, namely the IF and (oscillating) GIF neuron models. Their simplicity is useful in dissecting the relative contribution of very general dynamical mechanisms (such as passive and inductive membrane dynamics) to the emergence and properties of history-dependent processing. The IF neuron, described by a single dynamical variable with a single time constant, is a standard model of a passive membrane. The GIF neuron is a natural extension of the single variable IF model which includes an additional dynamical variable that varies linearly with voltage, and comes to represent the (linearized) net effect of voltage gated ionic currents. Of particular interest when dealing with temporally selective dynamical mechanisms is the oscillating GIF neuron, where the effect of the slow variable is to counteract voltage changes and the model dynamics is characterized by a pair of complex conjugate eigenvalues, thus exhibiting damped oscillatory responses to perturbing inputs. These simple linear models allowed the use of iterative formulae for calculating their response to input trains, and analytic expressions for the calculation of the intrinsic discriminability between pairs of input trains. The consequences and possible drawbacks of using these models are discussed in detail in the section “Limitations of the current approach”.

Both the IF and the GIF neuron exhibit history-dependent processing capabilities, which could allow the discrimination between different input trains even when they share the same average frequency or even the same constituent ISIs. In fact, history-dependent responses are a general feature of any dynamical system, and can be studied conveniently with any dynamic description of neuronal behavior (for example, through a set of differential equations). In this case, the neuron model does not implement a static input-output transformation, but one that is modulated by its state variables, which in turn reflect the previous history of stimuli and responses. The GIF model, though, is particularly sensitive to the precise timing of input stimuli, while the IF neuron discriminates better between inputs with different average frequencies (Fig. 5). This suggests that passive neurons are better suited to rate-based computation, while neurons with subthreshold oscillations are advantageous for information processing in a temporal coding scheme. Since context-dependent processing is observed even in these simple, linear models, we expect it to be a general feature of neuronal computation. Furthermore, we expect these history-dependent processing capabilities to become more complex and modulable as more realistic model neurons are considered, and as more time scales come into play.

One of the most important differences between the IF and the oscillating GIF model is that the intrinsic discriminability between two different input histories decreases exponentially with time for the IF neuron, while intrinsic oscillations result in an intrinsic discriminability that is an exponentially decreasing function of time multiplied by a sinusoid. This means that for certain input histories and neuron parameters, the instantaneous discriminability can reach its maximum a few u.t. after the last spike of the input train (Fig. 1A). Thus, when a certain neuron “decides” to transmit or block a certain piece of information depending upon the current context in the immediate past, intrinsic oscillations allow the reverberation of this information a few u.t. after the last spike of the input train which defines the context. Conversely, in passive neurons this information exponentially wanes.

Another important difference between oscillating and non-oscillating neurons regards the sensitivity to small differences in the input histories. For instance, when the discriminability between an accelerating and a decelerating input triplet with the same constituent ISIs is considered, the discriminability as a function of input ISI difference grows faster in the oscillating GIF neuron than in the purely passive IF neuron (Fig. 3B). Hence, if discriminability differences could be exploited as soon as they exceed a certain threshold, the oscillating GIF would exhibit an increased sensitivity in the discrimination between accelerating and decelerating triplets. This increase depends upon the input statistics though: the oscillating neuron discriminates better than the correspondent passive neuron between input triplets with frequency higher than a certain value, which decreases as the membrane rate constant 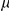 decreases (Fig. 3D). These results suggest that while a passive neuron can provide context-dependent processing capabilities in a broad frequency range (which nevertheless depends upon the membrane rate constant 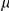), subthreshold oscillations enhance discriminability at high input frequencies and short ISI differences. Considering that discriminability differences below a certain threshold might not be physiologically exploitable for context-dependent processing, subthreshold oscillations increase the sensitivity between different input trains as a function of the difference between their constituent ISIs.

Finally, the theoretical framework provided by the History-Dependent Excitability concept allowed the assessment of the temporal extension of the intrinsic memory (section “Intrinsic discriminability between input trains that only differ in the their second spike time”). As the neuron receives successive EPSPs in a train, each EPSP drives the neuron's dynamical variables so that they come to reflect mainly the last input ISI, and the relative contribution of previous ISIs to the HDE gradually wanes. In these linear models the intrinsic discriminability of the first and second ISIs in a train decreases exponentially with the train length, and the rate of decay is not influenced by intrinsic oscillations. The exponential forgetting is a consequence of the small number of time scales interacting in these simple linear models (one for the IF, two for the GIF neuron). It would not be surprising if a subthreshold dynamics with multiple time scales might lead to a more gradual forgetting, for instance a power-law forgetting, as it is observed in multiple time scale synaptic models [54]. Indeed, a neuron model with a multiple time-scale current efficiently encodes input history over several time-scales [55]. Power-law forgetting curves could also arise from the interaction among many heterogeneous linear elements with exponentially decaying individual traces [56], [57].

The originality of this work lies in characterizing the discriminability properties of neurons based on their HDE trajectories, and not on their spiking output. This is an important shift in perspective because, as shown in Fig. 1, the HDE trajectory, but not the spiking output, determines how the neuron will respond to subsequent stimuli. Furthermore, our approach is quantitative and allows the evaluation of the history-dependent processing capabilities of different single-neuron dynamics, and their dependence upon input statistics and neuronal parameters.

Computer simulations allow the assessment of History-Dependent Excitability for arbitrary neuron models, and with arbitrary accuracy. In the absence of an analytical description for the HDE, it can be computed numerically by generating, in any moment in time, multiple branches of a given simulation, corresponding to different values of the conductance of an applied synaptic event. This technique allows the numerical measurement of the HDE, defined as the minimal conductance of a synaptic event capable of generating a spike, in a certain instant. Iterating this procedure over successive time steps allows the reconstruction of the HDE trajectory with arbitrary temporal resolution. The measurement of the HDE in living cells is constrained by the stability of the cell's properties, which will inevitably posit some limit upon the achievable resolution. Nevertheless, we believe that the experimental confirmation of the ideas exposed in this work is possible. In particular, an optimized dynamic clamp protocol [58] can be established, which could be used to probe the intrinsic discriminability as a function of input statistics, or simulated network state [59], with the minimum number of trials. These experiments will further our understanding of the relationships between single-cell properties, network state, and the temporal discriminability properties of single neurons. If the neuron considered exhibits chaotic dynamics in the subthreshold regime, the integral which defines the cumulative discriminability (1) might not converge. In this case, the memory about the previously received inputs, intended as the input-specific perturbation of the neuron's trajectory, persists for an infinite amount of time. This result is consistent with our interpretation of intrinsic discriminability as a measure of the amplitude and temporal extension of the transient mnemonic trace.

### Limitations of the current approach

Even if the computational mechanisms outlined in this work are general and apply to both living and model neurons, and might be relevant even to non-neural systems with history-dependent dynamics [60], the detailed analysis were carried out with linear models with one (in the case of the IF neuron) or two (for the GIF neuron) time scales. These models arise from a linearized approximation around the stable state, and are useful to describe the subthreshold response to weak inputs. Still, they might not be adequate to study neuronal networks that can operate in different regimes, or that exhibit local or global transitions between up and down states [9], [61], [62]. For instance, when a real neuron (or a non-linear neuron model) is clamped to different holding voltages with a proper injected DC current, the jacobian matrix resulting from linearization around this new stable state is in general different from that obtained at equilibrium and in the absence of external currents. This implies that its resonant and passive properties will strongly depend upon the voltage the neuron is held at, or, in more physiological conditions, on the state of the subset of the circuit impinging on it [63]. We predict that the computational consequences of History-Dependent Excitability could be especially relevant in the down state, where the effective membrane time constant is slow and the membrane potential is far from threshold. In particular, we can speculate that the proposed mechanisms might contribute to the selection of the subset of neurons which will take part in the up state, depending upon the present context.

Another simplification adopted is the spike generation mechanism, implemented as a fixed voltage threshold in the IF and GIF models. It is possible that in certain conditions this simplification might provide a biased estimate of the HDE (which depends on both the synaptic dynamics and the spike generation mechanism). For instance, the interplay between the spike generation mechanism and the synaptic filtering properties can affect single-neuron processing, especially at high input rates [64]. Moreover, neurons that display intrinsic oscillations are likely to be dynamically close to an Andronov-Hopf bifurcation, hence their firing threshold might be better described by a curved manifold rather than by a fixed voltage threshold. This issue will be addressed in future work, where the influence of the spike generation mechanisms and its interplay with synaptic kinetics will be assessed by using computational models of increasing realism.

In this work we have assessed the influence of subthreshold damped oscillations arising from intrinsic neuronal dynamics upon information discrimination. In several brain areas and functional conditions sustained oscillations of the membrane potential have been observed. These oscillations are thought to arise mainly through network mechanisms [30], even if intrinsic oscillatory dynamics at the single cell level can also play a role [28]. Since membrane oscillations arising from network mechanisms are the result of external oscillatory input currents, they do not affect intrinsic discriminability in the linear subthreshold approximation considered here (see Section “Discriminability between pairs of input trains”). Sustained subthreshold oscillations can also arise from intrinsic mechanisms in the presence of a subthreshold limit cycle or strange attractor. In this case intrinsic discriminability is expected to depend upon the state vector at the time of each applied synaptic potential, and its characterization can be accomplished through numerical analysis.

In this article we considered an idealized scenario in which the neuron sits at its stable point, receives a train of instantaneous EPSPs with constant amplitude, and it is then free to evolve according to its intrinsic dynamics alone. The adoption of linear models guarantees that the presented results also hold if the input histories include fluctuating currents (“frozen noise”), as long as they are equal among different input histories. Furthermore, intrinsic discriminability does not depend upon the neuron state at the moment of input arrival in linear models. Nevertheless, it is not clear whether intrinsic discriminability could still serve as a reliable indicator of the current context in a complex, non-linear neuron as the input histories include unknown time-varying components. From a dynamical perspective, the consistent encoding of incoming information could still be achieved if the neuron state is driven to a restricted portion of its state space before receiving its temporally-structured inputs. This task could be accomplished by network oscillations [30], [65]: periodic inhibition could format the continuous stream of incoming synaptic events in blocks of fixed temporal duration, by driving the neuron to a restricted portion of its state space at each peak of the total inhibitory drive [66]. This periodic “reset” would allow the consistent contextualization of the EPSPs on a cycle-by-cycle basis. Periodic excitation could play a similar role. For networks operating in an asynchronous, irregular firing regime, results from random dynamical system theory [67] reveal that in certain conditions a neuron injected with a pseudo-random stimulus will soon forget its initial state and converge to a stochastic, time-varying attractor [68], [69]. Hence, temporally structured inputs time-locked to the stochastic attractor might be consistently encoded through the proposed mechanism even in non-linear neurons. In this case, the transient memory trace induced by the external stimulus would gradually wane as the neuron trajectory converges to the stochastic attractor corresponding to a given noise realization. Nevertheless, more work is needed in the field in order to clarify the interplay between sensitivity and reliability in neuronal network dynamics [70]–[72].

Recently, Branco *et al.* proposed a new mechanism for the discrimination of spatiotemporal inputs based upon NMDA receptor dynamics, along with the impedance gradient resulting from dendritic tapering [42]. In their experiments, different spatiotemporal patterns of synaptic potentials evoked on a dendrite via two-photon glutamate uncaging resulted in PSPs of different amplitude at the soma. Their work shows that neuronal morphology and non-linear receptor dynamics, which were not taken into account here, are important features in determining the spatiotemporal selectivity of neurons and are surely expected to add a layer of complexity to the history-dependent discriminability properties we described. On the other hand, the neurons studied by Branco *et al.* seem to lack prominently active dendrites, and the kind of selectivity they report can be summarized as a consistently greater somatic depolarization in response to centripetal, rather than centrifugal, patterns of stimulation. Our work suggests an important role for active dendritic properties in the discrimination of spatiotemporal inputs. In particular, the distribution of active ion channels could be a key ingredient in achieving greater flexibility and modularity in the discrimination of spatiotemporal inputs at the single-cell level.

One last remark regards the definition of History-Dependent Excitability as the minimal excitatory synaptic strength capable of firing the neuron. In the translation from the state vector to a scalar, spike-related measure of the neuron's propensity to firing, we defined HDE as the minimal excitatory synaptic strength capable of firing the neuron. While such a translation is useful for the comparison between different neuron types, the exact definition we propose might sound arbitrary. For instance, neurons receiving an intense barrage of EPSPs and IPSPs might fire mostly because of a reduction in the total inhibitory conductance, rather than because of an increment in the total excitatory drive [73]. A generalization of the HDE that takes into account post-inhibitory rebound spiking and more general threshold manifolds will be necessary to gain insight into this issue. While the exact definition of HDE is somewhat arbitrary, the general concept presented is powerful enough to foster the study of single-neuron discrimination properties, which has been largely disregarded in the literature.

### Consequences at the network level

The presence of an intrinsic memory in each processing unit is likely to have profound consequences at the network level, and to foster the emergence of complex functions in biological circuits. It is now widely accepted that network function is not entirely specified by the network connectivity, but depends upon its neuromodulatory state, and its history of activity and afferent inputs (see, for example, [59], [74], [75], reviewed in [76]). Sometimes the same network can produce the same output in different network states, which are only revealed if specific electrophysiological or pharmacological manipulations are applied to the network [74], [77]. On the other hand, the state of the network shapes the input-output transformation performed by its constituent neurons, by setting the statistics of the synaptic currents that a representative neuron in the network receives [59], [63], [78], [79]. We believe that the presence of context-dependent processing capabilities at the single-cell level, or even down to the dendritic domain level, coupled with the domain-specific interneuron innervation [80], is a key feature in the emergence of multifunctionality in neuronal networks.

The stimulation of some, but not all, cortical neurons can result in global effects at the level of the whole animal, such as behaviorally reportable perceptions, the generation of motor actions and changes in the global brain state (recently reviewed in [81]). These reports, together with the observation of sparse and abstract representations in high order brain areas, suggest an influential role for individual neurons in brain function which has been largely disregarded so far. In particular, how downstream areas can read out a sparse code is still not known. However, they cannot rely solely upon spatial summation and coincidence detection, but must use some efficient mechanisms for the recognition of the identity of the activated neurons. The presence of strong synaptic connections [47], [49], sometimes targeting areas in the postsynaptic neurons which are key for the generation of action potentials (as in the case of mossy fibers innervation of Purkinje cells [82]), is an important factor in achieving reliable information transfer. Furthermore, the observation of cell-specific firing patterns in widely different systems [83], [84] hints at the presence of a multiplexed code where the neuron identity (“who”) could be transmitted together with the circumstantial message (“what”) [85]. The presence of complex history-dependent processing capabilities with fine temporal sensitivity at the subcellular level is likely a key feature for the interpretation and transformation of sparse neuronal codes.

Traditionally, neuronal network computations have been interpreted within a static framework. For instance, in associative neural networks, the synaptic weights determine the set of attractors toward which any initial network state will evolve, after a transient which is thought to bear no relevant information [86]. However, working memory studies reported that individual neurons can shift their tuning from a purely stimulus-driven sensitivity to the encoding of prospective actions [87]–[91]. These experiments have shown that working memory cannot be clearly disentangled from perception, expectation, prediction, and other cognitive computations, since they rely upon largely overlapping neuronal networks. Instead, it seems that transient dynamics in the brain underlie the joint representation of memory clues and what the brain does with them (see also [92]).

In the last few years new theoretical paradigms have been proposed, which are better suited to the interpretation of transient and dynamical computation in neuronal networks (reviewed in [93]–[98]). In particular, some authors have proposed that the variety of time scales involved in neuronal network dynamics, and their distributed nature among many constituent neurons and synapses, form a general purpose computing substrate from which the useful information can be extracted at any given time by properly trained read-out neurons [99]–[101]. In these models (commonly referred to as “state-dependent networks” or “liquid state machines”) the distributed synaptic dynamics encodes input history in an analogous way as we discussed in this paper for a single neuron: each input to the network will have an effect that depends upon the previous network history. In contrast to the linear, few dimensional single-cell models considered in this work, liquid state machines retain some information about past stimuli for periods of time that are much longer than the slower time scale in the system. This behavior is due to their lack of attractors and “edge of chaos” dynamics [102], and to the reverberation of activity through positive feedback. These characteristics make them exquisitely sensitive to small perturbations, whose contributions linger for a long time in the network dynamics.

We believe that the contributions of this work to the theory of state-dependent networks are twofold. On the one hand, the complex dynamical entities in these models are the synaptic connections, while the intrinsic neuronal dynamics is commonly reduced to a purely passive description. We believe that intrinsic neuronal processes also play a part in determining the complex dynamics of neural microcircuits: as we showed in this paper, the intrinsic properties determine the evolution of neuronal excitability as a function of the previous history, and will eventually determine whether future stimuli will result in a post synaptic spike or not. On the other hand, when the network state is considered and related to network performance, only the voltage variable of each neuron and the strength of each synapse are taken into account, while internal variables are disregarded [101]. This work shows that the information contained in single-neuron dynamics is not only that which is transmitted to other neurons in the network through spike-mediated synaptic transmission, but also includes the intrinsic subthreshold dynamics which affects the way a neuron responds to incoming stimuli. Hence, an assessment of their information processing capabilities should take into account all the dynamical variables of the model (synaptic and intrinsic), this being the only level of description that unambiguously defines the microcircuit high-dimensional input-output mapping to an arbitrary spatiotemporal stimulus.

In addition to this, there is a substantial heterogeneity in the intrinsic properties of individual cells in several brain regions, which goes beyond the categorization in neuronal subtypes commonly accepted in the literature [103]. Nevertheless, most theoretical approaches to neuronal network dynamics have considered the individual cells as homogeneous, or divided in two or few homogeneous populations (for example, excitatory and inhibitory populations [104]). More recent studies have revealed that neuronal heterogeneity might not be just an epiphenomenon, but might serve specific computational purposes, like the improvement of information representation in a population code [105], [106], or the generation of complex dynamics in recurrent networks [107]. Furthermore, intrinsic neuronal properties and synaptic connectivity are expected to be correlated, given that intrinsic oscillations bias weight dynamics under spike-timing dependent synaptic plasticity [108]. Hence we strongly encourage the inclusion of a realistic level of heterogeneity in the intrinsic properties of individual cells, as it is likely to highlight new important roles for the wide diversity of intrinsic and synaptic properties in the nervous system.

## Methods

### Neuron models

The first neuron model we consider is the integrate and fire (IF), described by a single linear differential equation:
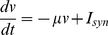
(9)


The model is endowed with an after-spike reset mechanism, so that when 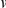 crosses a threshold 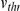 from below a spike is emitted and the membrane potential is reset to a value 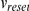, and kept there for a refractory time 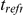. Since this work focuses on subthreshold dynamics, the spike generation mechanism is disregarded. In its normal form (where time has been properly scaled) this model is described by a single parameter 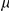, which is the rate of the exponential decay to the rest state in the absence of stimulation (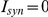). The canonical IF model that we used for most of the paper has a decay rate 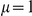, but this parameter has been varied in the simulations represented in Figs. 3D,F; 4; 6B,E.

Another simple model that linearly describes the subthreshold dynamics is the Generalized Integrate and Fire (GIF) model. This model includes an additional dynamical variable, which represents the linearized effect of voltage-gated ion currents, and is described by the following equations:
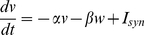
(10)

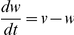



with the same after-spike resetting as in the IF model for the 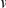 variable, while no reset is applied to the additional dynamical variable 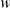. In this work only the subthreshold dynamics has been considered, and the spike generation mechanism has not been taken into account. In its normal form the model is parameterized by an effective leak 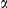 and an effective coupling between the two variables 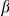. If 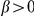 the 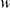 variable opposes voltage change, providing a negative feedback and reproducing the effect of resonance currents such as a 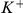 or 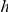 current. In this case, the coupled dynamics can exhibit subthreshold resonance and even damped oscillations. Conversely, if 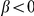 the 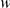 variable amplifies voltage change, providing a positive feedback and mimicking the effect of amplifying currents such as a 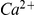 or 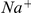 current. The system (10) has proven particularly useful in studying neuronal intrinsic oscillations [31], [32], [109]: in a certain parameter regime, it is mathematically equivalent to a damped linear oscillator, and thus constitutes an analytically amenable model for the description of neuronal intrinsic oscillations, i.e., oscillations generated by intrinsic ionic mechanisms as the activation of a resonant current or the inactivation of an amplifying current [29].

The canonical GIF model that we used for most of the paper has 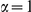 and 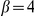, resulting in complex conjugate eigenvalues 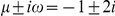 (see Text S1), but these parameters have been varied in the simulations represented in Figs. 3D-F; 4; 6A,B,E,F; 7. The presence of complex conjugate eigenvalues denotes an oscillatory behavior with an angular frequency equal to 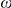, the imaginary part of the eigenvalues, while the real part 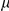 determines the rate of decay to the rest state. Given the wide frequency range of intrinsic oscillations observed in mammalian brains, which spans at least two order of magnitude (from 0.5 Hz until 50 Hz [29]), we preferred to keep our models dimensionless.

For the sake of simplicity, and for carrying out analytical calculations, we consider
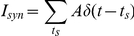



This approximation reduces the synaptic dynamics to an instantaneous and voltage independent shift in the voltage variable. If the considered intrinsic dynamics is slow with respect to the synaptic dynamics, and if the membrane voltage stays in a narrow range with respect to the distance from the synaptic reversal potential, this approximation is reasonable. The model trajectories have been calculated with iterative formulae (see Text S1).

### Generation of random trains of synaptic events

In the simulations, random trains of synaptic events have been generated by concatenating two or more ISIs generated from an exponential distribution, using the Matlab routine “random”. For each value of the ranged parameters, 1000 different trials have been simulated, and the corresponding results have been represented in Figs. 6C,D and 7. In each block of simulations the random number generator has been initialized with a different seed in order to avoid spurious correlations between the random realizations in different trials.

## Supporting Information

Text S1 Appendix.
(PDF)
