# Supplementary material for: History-Dependent Excitability as a Single-Cell Substrate of Transient Memory for Information Discrimination
Source: PLoS One. 2010 Dec 28;5(12):e15023. doi: 10.1371/journal.pone.0015023 (PMC3010997; doi:10.1371/journal.pone.0015023)
Supplement: Text S1 — Appendix. (PDF) [file pone.0015023.s001.pdf]

Appendix

Integration of the neuron models

The differential equations describing the IF and GIF models can be solved analytically given the initial conditions of the dynamical variables.

IF model

In the absence of synaptic input ($I_{syn} = 0$), the solution to (9) reads

$$v(t) = v_0 \mathrm{e}^{-\mu t}$$

where v_0 is the initial condition of the voltage variable at time $t = 0$. Hence, in the absence of post synaptic spikes (see Methods), the response of the model neuron to a train of synaptic events at times $(t_0^s, t_1^s, \ldots, t_{n-1}^s)$ can be calculated by iterating the following map:

$$v_i = (v_{i-1} + A)\mathrm{e}^{-\mu \mathrm{ISI_i}}$$

for $i = 1, \ldots, n-1$, where $\mathrm{ISI}_i = t_i^s - t_{i-1}^s$, A is the synaptic strength (assumed for simplicity to be equal among synaptic events), and v_i is the voltage variable just before the arrival of the synaptic event at time t_i^s. The voltage variable after the last spike of the train is calculated as

$$v_n = (v_{n-1} + A)$$

and will be used to calculate the discriminability (see the next section). In all our simulations we set the initial condition for the v variables at the rest state ($v_0 = 0$).

GIF model

In the absence of synaptic input ($I_{syn} = 0$), the subthreshold dynamics (10) has as a general solution

$$\vec{s}(t) = \mathrm{e}^{-\mu t}(c_1 \mathrm{e}^{i\omega t}\vec{u}_1 + c_2 \mathrm{e}^{-i\omega t}\vec{u}_2) \qquad \text{(A-1)}$$

where $\mu = \frac{\alpha+1}{2}$ and $\omega = \frac{\sqrt{4\beta-(1-\alpha)^2}}{2}$. The parameter μ determines the membrane time constant, while the parameter ω is the oscillation frequency (in 1/rad), related to the intrinsic period of oscillations $T = \frac{2\pi}{\omega}$. We chose to fix μ at a physiological value ($\mu = 1$). With this choice, $\omega = \sqrt{\beta}$.

The coefficients c_1 and c_2 depends on the initial conditions and can be easily derived analytically. Considering that $\vec{u}_1 = \begin{pmatrix} 1+\lambda_1 \\ 1 \end{pmatrix}$ and $\vec{u}_2 = \begin{pmatrix} 1+\lambda_2 \\ 1 \end{pmatrix}$ are eigenvectors of the system (10) associated with the eigenvalues $\lambda_1 = -\mu + i\omega$ and $\lambda_2 = -\mu - i\omega$, respectively, by evaluating (A-1) at $t = 0$ and projecting on the v and w axis one obtains:

$$\begin{aligned} v_0 &= c_1(1+\lambda_1) + c_2(1+\lambda_2) \\ w_0 &= c_1 + c_2 \end{aligned}$$

where v_0 and w_0 are the general initial conditions for the voltage and the slow variable at $t = 0$. By solving for c_1 and c_2 and substituting $\mathrm{e}^{i\omega} = \cos(\omega) + i\sin(\omega)$ one obtains the following expressions for the free evolution of the system (10) given the initial condition (v_0, w_0):

$$
\begin{aligned}
v(t) &= \mathrm{e}^{-\mu t}\left(v_0\cos(\omega t) + \frac{v_0(1-\mu) - w_0(1-2\mu+\mu^2+\omega^2)}{\omega}\sin(\omega t)\right)\\
w(t) &= \mathrm{e}^{-\mu t}\left(w_0\cos(\omega t) + \frac{v_0 - w_0(1-\mu)}{\omega}\sin(\omega t)\right)
\end{aligned}
$$

Hence, if we model the EPSPs as instantaneous pulses along the v axis, it is possible to compute analytically the response to a train of presynaptic pulses. In the absence of post synaptic spikes (see Methods), the response of the model neuron to a train of synaptic events at times $(t_0^s, t_1^s, \ldots, t_{n-1}^s)$ can be calculated by iterating the following map:

$$
\begin{aligned}
v_i &= \mathrm{e}^{-\mu \mathrm{ISI_i}}\left((v_{i-1}+A)\cos(\omega \mathrm{ISI_i}) + \frac{(v_{i-1}+A)(1-\mu) - w_{i-1}(1-2\mu+\mu^2+\omega^2)}{\omega}\sin(\omega \mathrm{ISI_i})\right)\\
w_i &= \mathrm{e}^{-\mu \mathrm{ISI_i}}\left(w_{i-1}\cos(\omega \mathrm{ISI_i}) + \frac{v_{i-1}+A - w_{i-1}(1-\mu)}{\omega}\sin(\omega \mathrm{ISI_i})\right)
\end{aligned}
$$

for $i = 1, \ldots, n-1$, where $\mathrm{ISI}_i = t_i^s - t_{i-1}^s$, A is the synaptic strength (assumed to be equal among synaptic events), and (v_i, w_i) are the dynamical variables just before the arrival of the synaptic event at time t_i^s. The voltage variable after the last spike of the train is calculated as

$$
v_n = (v_{n-1} + A)
$$

and will be used to calculate the discriminability (see the next section). In all our simulations we set the initial conditions at the rest state $(v_0 = 0, w_0 = 0)$.

Analytical derivation of the discriminability

We define the neuronal excitability in any moment in time as the minimal synaptic strength capable of firing the neuron. The instantaneous intrinsic discriminability between two input histories is then defined, in any moment in time, as the square difference between the excitabilities corresponding to the two input histories. That is,

$$
\mathcal{D}_{i,j}(t) = \left(g^i_{thr}(t) - g^j_{thr}(t)\right)^2 = \left(v^j(t) - v^i(t)\right)^2 \tag{A-2}
$$

where the last equality is due to the fact that in these linear models the firing threshold depends upon the voltage variable v only. The main measures of intrinsic discriminability we considered are the maximal instantaneous discriminability (maximal value of (A-2)) and the cumulative discriminability, defined as the integral in time from zero to $+\infty$ of the instantaneous discriminability:

$$
\mathcal{D}^{\infty}_{i,j} = \int_0^{\infty}\left(g^i_{thr}(t) - g^j_{thr}(t)\right)^2 dt = \int_0^{\infty}\left(v^j(t) - v^i(t)\right)^2 dt
$$

The instantaneous and the cumulative discriminability can be computed analytically for the IF and GIF neuron, given the initial conditions for the dynamical variables.

Analytical derivation of the discriminability: IF neuron

For the IF model neuron the instantaneous discriminability can be written as a function of the initial conditions for the voltage variable v_0^i and v_0^j, corresponding to the neuron's state after input history $\mathcal{H}_i$ and $\mathcal{H}_j$, respectively:

$$\mathcal{D}_{i,j}(t) = \left(v^j(t) - v^i(t)\right)^2 = (v_0^j - v_0^i)^2 \mathrm{e}^{-2\mu t}$$

This expression integrated from zero to $+\infty$ gives the cumulative discriminability:

$$\mathcal{D}_{i,j}^{\infty} = \int_0^{\infty} \left(v^j(t) - v^i(t)\right)^2 dt = (v_0^j - v_0^i)^2 \int_0^{\infty} \mathrm{e}^{-2\mu t} dt = \frac{(v_0^j - v_0^i)^2}{2\mu}$$

Analytical derivation of the discriminability: GIF neuron

For the GIF model neuron the instantaneous discriminability can be written as a function of the initial conditions for the dynamical variables (v_0^i, w_0^i) and (v_0^j, w_0^j), corresponding to the neuron's state after input history $\mathcal{H}_i$ and $\mathcal{H}_j$, respectively:

$$\mathcal{D}_{i,j}(t) = \left(g_{thr}^i(t) - g_{thr}^j(t)\right)^2 = \mathrm{e}^{-2\mu t} \left[(a_j - a_i)\cos(\omega t) + (b_j - b_i)\sin(\omega t)\right]^2$$

where we set $a_{i,j} = v_0^{i,j}$ and $b_{i,j} = \frac{v_0^{i,j}(1-\mu) - w_0^{i,j}(1-2\mu+\mu^2+\omega^2)}{\omega}$. Calculating the square product of the bracketed expression and applying some trigonometric equalities one obtains:

$$\mathcal{D}_{i,j}(t) = \mathrm{e}^{-2\mu t} \left[(a_j - a_i)^2 \frac{1+\cos(2\omega t)}{2} + (b_j - b_i)^2 \frac{1-\cos(2\omega t)}{2} + (a_j - a_i)(b_j - b_i)\sin(2\omega t)\right]$$

and finally

$$\mathcal{D}_{i,j}(t) = \mathrm{e}^{-2\mu t} \left(\frac{a^2+b^2}{2} + \frac{a^2-b^2}{2}\cos 2\omega t + ab\sin 2\omega t\right)$$

with $a = a_j - a_i$ and $b = b_j - b_i$.

The above expression integrated from zero to $+\infty$ gives the cumulative discriminability:

$$\mathcal{D}_{i,j}^{\infty} = \frac{a^2+b^2}{4\mu} + \frac{\mu(a^2-b^2) + 2\omega ab}{4(\mu^2+\omega^2)}$$

where the indefinite integrals

$$\int \mathrm{e}^{ax}\cos bx = \frac{b}{a^2+b^2}\mathrm{e}^{ax}\sin bx + \frac{a}{a^2+b^2}\mathrm{e}^{ax}\cos bx$$
$$\int \mathrm{e}^{ax}\sin bx = \frac{a}{a^2+b^2}\mathrm{e}^{ax}\sin bx - \frac{b}{a^2+b^2}\mathrm{e}^{ax}\cos bx$$

have been used. Thus, it is a quadratic function in $\Delta v = v_j - v_i$ and $\Delta w = w_j - w_i$, whose orientation and eccentricity are determined by the neuron's parameters μ and ω.

Average discriminability in the presence of Gaussian inputs

The average discriminability in the presence of Gaussian inputs can be calculated as

$$E[\mathcal{D}] = \int_{-\infty}^{0}\int_{-\infty}^{0} \mathcal{D}(i,j) \frac{4}{\mathrm{erfc}\left(\frac{m_i}{\sqrt{2}\sigma}\right)\mathrm{erfc}\left(\frac{m_j}{\sqrt{2}\sigma}\right)2\pi\sigma^2} \mathrm{e}^{-\frac{(t_i-m_i)^2}{2\sigma^2}} \mathrm{e}^{-\frac{(t_j-m_j)^2}{2\sigma^2}} dt_i dt_j$$

where the factors $\text{erfc}\left(\frac{m_i}{\sqrt{2}\sigma}\right)$ and $\text{erfc}\left(\frac{m_j}{\sqrt{2}\sigma}\right)$ take into account the fact that ISIs cannot be non-positive. The standard deviations are assumed equal for both distributions for simplicity. If $\mathcal{D}(i,j) = \mathcal{D}_{IF}(i,j)$ solving yields:

$$
\begin{aligned}
E[\mathcal{D}_{IF}] = &\left(A^2 \left(\mathrm{e}^{2\mu\left(\mathrm{m_i}+\mu\sigma^2\right)}\text{erfc}\left(\frac{\mathrm{m_j}}{\sqrt{2}\sigma}\right)\text{erfc}\left(\frac{\left(2\mu+\frac{\mathrm{m_i}}{\sigma^2}\right)\sigma}{\sqrt{2}}\right) - \right.\right.\\
&2\mathrm{e}^{\mu\left(\mathrm{m_i}+\mathrm{m_j}+\mu\sigma^2\right)}\text{erfc}\left(\frac{\left(\mu+\frac{\mathrm{m_i}}{\sigma^2}\right)\sigma}{\sqrt{2}}\right)\text{erfc}\left(\frac{\left(\mu+\frac{\mathrm{m_j}}{\sigma^2}\right)\sigma}{\sqrt{2}}\right) + \\
&\left.\left.\mathrm{e}^{2\mu\left(\mathrm{m_j}+\mu\sigma^2\right)}\text{erfc}\left(\frac{\mathrm{m_i}}{\sqrt{2}\sigma}\right)\text{erfc}\left(\frac{\left(2\mu+\frac{\mathrm{m_j}}{\sigma^2}\right)\sigma}{\sqrt{2}}\right)\right)\right) \Big/ \left(2\mu\text{erfc}\left(\frac{\mathrm{m_i}}{\sqrt{2}\sigma}\right)\text{erfc}\left(\frac{\mathrm{m_j}}{\sqrt{2}\sigma}\right)\right)
\end{aligned}
$$

In the case of the GIF neuron we further assumed $m_i = m_j = m$ for simplicity, obtaining

$$
\begin{aligned}
E[\mathcal{D}_{GIF}] = &\frac{A^2\mathrm{e}^{2m\mu+\sigma^2\left(\mu^2-5\omega^2\right)}}{8\mu\omega^2\left(\mu^2+\omega^2\right)\text{erfc}\left(\frac{m}{\sqrt{2}\sigma}\right)^2}\left(2\mathrm{e}^{4\sigma^2\omega^2}\left(\mathrm{e}^{-2i\left(m+\mu\sigma^2\right)\omega}\mu(-1+\mu-i\omega)^2(\mu+i\omega)\text{erfc}^2\left(\frac{m+\sigma^2(\mu-i\omega)}{\sqrt{2}\sigma}\right) - \right.\right.\\
&2\left((-1+\mu)^2+\omega^2\right)\left(\mu^2+\omega^2\right)\text{erfc}\left(\frac{m+\sigma^2(\mu-i\omega)}{\sqrt{2}\sigma}\right)\text{erfc}\left(\frac{m+\sigma^2(\mu+i\omega)}{\sqrt{2}\sigma}\right) + \\
&\left.\mathrm{e}^{2i\left(m+\mu\sigma^2\right)\omega}\mu(\mu-i\omega)(-1+\mu+i\omega)^2\text{erfc}^2\left(\frac{m+\sigma^2(\mu+i\omega)}{\sqrt{2}\sigma}\right)\right) + \\
&2\text{erfc}\left(\frac{m}{\sqrt{2}\sigma}\right)\left(2\mathrm{e}^{\sigma^2\left(\mu^2+5\omega^2\right)}\left((-1+\mu)^2+\omega^2\right)\left(\mu^2+\omega^2\right)\text{erfc}\left(\frac{m+2\mu\sigma^2}{\sqrt{2}\sigma}\right) - \right.\\
&\mathrm{e}^{\sigma^2\left(\mu^2+3\omega^2\right)}\mu\left(\mathrm{e}^{-2i\left(m+2\mu\sigma^2\right)\omega}(-1+\mu-i\omega)^2(\mu+i\omega)\text{erfc}\left(\frac{m+2\sigma^2(\mu-i\omega)}{\sqrt{2}\sigma}\right) + \right.\\
&\left.\left.\left.\mathrm{e}^{2i\left(m+2\mu\sigma^2\right)\omega}(\mu-i\omega)(-1+\mu+i\omega)^2\text{erfc}\left(\frac{m+2\sigma^2(\mu+i\omega)}{\sqrt{2}\sigma}\right)\right)\right)\right)
\end{aligned}
$$

Although the imaginary unit i appears in the above expression, it always evaluates to real values if the model parameters are real.
